# Role of nerve–muscle interactions and reactive oxygen species in regulation of muscle proteostasis with ageing

**DOI:** 10.1113/JP274336

**Published:** 2017-09-10

**Authors:** Aphrodite Vasilaki, Arlan Richardson, Holly Van Remmen, Susan V. Brooks, Lisa Larkin, Anne McArdle, Malcolm J. Jackson

**Affiliations:** ^1^ Department of Musculoskeletal Biology, MRC Arthritis Research UK Centre for Integrated Research into Musculoskeletal Ageing (CIMA), Institute of Ageing and Chronic Disease University of Liverpool Liverpool UK; ^2^ Department of Geriatric Medicine and the Reynolds Oklahoma Center on Aging Oklahoma University Health Science Center Oklahoma City OK USA; ^3^ Oklahoma City VA Medical Center Oklahoma City OK USA; ^4^ Aging and Metabolism Division Oklahoma Medical Research Foundation Oklahoma City OK USA; ^5^ Department of Molecular and Integrative Physiology University of Michigan Ann Arbor MI USA; ^6^ Department of Biomedical Engineering University of Michigan Ann Arbor MI USA

**Keywords:** Cu/Zn superoxide dismutase, frailty, neuromuscular homeostasis, oxidative stress, sarcopenia

## Abstract

Skeletal muscle ageing is characterised by atrophy, a deficit in specific force generation, increased susceptibility to injury, and incomplete recovery after severe damage. The hypothesis that increased generation of reactive oxygen species (ROS) *in vivo* plays a key role in the ageing process has been extensively studied, but remains controversial. Skeletal muscle generates ROS at rest and during exercise. ROS can cause oxidative damage particularly to proteins. Indeed, products of oxidative damage accumulate in skeletal muscle during ageing and the ability of muscle cells to respond to increased ROS becomes defective. The aim of this review is to examine the evidence that ROS manipulation in peripheral nerves and/or muscle modifies mechanisms of proteostasis in skeletal muscle and plays a key role in initiating sarcopenia.

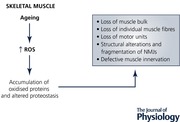

**Abbreviations**
CMA: chaperone‐mediated autophagy
Cu/ZnSOD: copper, zinc superoxide dismutase
EMG: electromyography
HSP: heat shock protein
LAMP‐2A: lysosome‐associated membrane protein type 2A
MA: macroautophagy
MnSOD: manganese superoxide dismutase
mtROS: mitochondrial ROS
NFκB: nuclear transcription factor kappa B
NMJs: neuromuscular junctions
ROS: reactive oxygen species
SOD: superoxide dismutase
*Sod1*: copper, zinc superoxide dismutase
WT: wild‐type

## Introduction

A 30–50% loss of muscle mass occurs between the ages of 50 and 80 years (Fried *et al*. 2001; Bortz, 2002; Espinoza & Walston, 2005; Cesari *et al*. 2006) that impacts profoundly on the quality of life of older people resulting in a reduced ability to carry out everyday tasks and increased likelihood of falling. All individuals lose muscle mass and develop age‐related muscle weakness (termed sarcopenia when it reaches clinically relevant severity), although some individuals are more prone to sarcopenia. The underlying mechanisms are unclear, but an individual's initial muscle mass appears to be a critical factor influencing the risk of developing sarcopenia. For example, veteran athletes lose muscle mass at the same rate as sedentary individuals, but have a high peak muscle mass in earlier life and reach the threshold for poor function at a later age than sedentary individuals (Pearson *et al*. 2002). Muscle mass is dictated by the number of muscle fibres and the size of the fibres. The decline in muscle mass and strength in people after the age of ∼50 appears primarily due to loss of muscle fibres with weakening of the remaining fibres (Marzetti *et al*. 2009). For example, in humans, Lexell *et al*. (1988) reported 40% fewer fibres in the vastus lateralis quadriceps muscles of older individuals (Lexell *et al*. 1988).

Data clearly indicate that in ageing man and rodents, loss of motor neurons accompanies the loss of muscle fibres (Campbell *et al*. 1973; Brown *et al*. 1988; Einsiedel & Luff, 1992; Larsson & Ansved, 1995), but whether motor neuron loss is a cause or a consequence of muscle fibre deficits has not been definitively established due largely to the lack of available techniques to directly count motor units (a motor unit consists of a single α‐motor neuron and all of the muscle fibres it innervates) in humans. Measurements have been limited to *post mortem* anatomical estimates or estimates based on electromyography (EMG). *Post mortem* anatomical studies have shown that people aged 75 years have 30% fewer motor neurons supplying the muscles of the lower limbs compared with young adults (Kawamura *et al*. 1977; Tomlinson & Irving, 1977; Mittal & Logmani, 1987). One of the first studies to demonstrate motor unit loss during healthy ageing using EMG was by Campbell *et al*. (1973), who recorded evoked potentials and compared these with maximum M‐waves to estimate that there were 50% fewer motor units in the extensor digitorum brevis in people above 60 years. Brown *et al* (1988) also demonstrated that subjects over 60 years of age have approximately half the numbers of motor units of subjects less than 60 years of age (Brown *et al*. 1988) and more recently, using intramuscular and surface EMG signals from the vastus lateralis during voluntary contractions, Piasecki *et al*. demonstrated that the total number of motor units in muscles from older individuals (above 65 years) was between 50% and 60% lower compared to those in young (Piasecki *et al*. 2016b). The range of techniques available to estimate motor unit numbers in humans and their limitations have been reviewed elsewhere (Piasecki *et al*. 2016a).

Several studies have also reported swollen, segmental demyelinated and remyelinated axons in peripheral nerve of old animals and humans (Sharma *et al*. 1980; Grover‐Johnson & Spencer, 1981; Adinolfi *et al*. 1991; Verdu *et al*. 2000) and such neuronal changes have been proposed to play a major role in the age‐related loss of muscle mass and function (Delbono, 2003). Neuromuscular junctions (NMJs) in muscle fibres of old mice show a variety of alterations, including axonal swellings, sprouting, synaptic detachment, withdrawal of axons from postsynaptic sites and fragmentation of the acetylcholine receptors (AChRs) (Valdez *et al*. 2010; Chai *et al*. 2011; Vasilaki *et al*. 2016).

### Reactive oxygen species and their role in the loss of skeletal muscle mass during ageing

Regulated changes in reactive oxygen species (ROS) formation are important in signalling to maintain normal physiological processes in all cells including muscles and neurons. The main physiological mechanism by which cells regulate ROS activities (and hence protect against oxidative damage) is by modification of the expression and activities of regulatory enzymes such as manganese superoxide dismutase (MnSOD), copper, zinc superoxide dismutase (Cu/ZnSOD), catalase, glutathione peroxidases and haem oxygenase‐1 (Powers & Jackson, 2008). Acute increases in ROS generation in skeletal muscle lead to activation of a number of redox‐sensitive transcription factors, including nuclear transcription factor kappa B (NFκB) and activator protein‐1 (Vasilaki *et al*. 2006b; Gomez‐Cabrera *et al*. 2008) with the subsequent increased expression of antioxidant defence enzymes such as superoxide dismutase (SOD) and catalase and cytoprotective proteins such as heat shock proteins (HSPs) (McArdle *et al*. 2001; Vasilaki *et al*. 2006b). Motor neurons also have the capacity to upregulate some of these cytoprotective proteins in response to exogenous reactive oxygen and nitrogen species (Bishop *et al*. 1999).

ROS activities in many tissues increase with age and there is evidence that increased ROS generation may be involved in several age‐related pathologies. In skeletal muscle, *in vitro* and *in vivo* studies have provided evidence of an age‐related increase in ROS production (Vasilaki *et al*. 2006a; Palomero *et al*. 2013). Increased ROS production can lead to changes in the redox state of muscle cells with potentially serious effects on muscle such as cumulative damage to cellular macromolecules including lipids in cell membranes, DNA, and subcellular membranes and structures. A major effect of aberrant ROS generation is oxidative damage to proteins (Levine & Stadtman, 2001; Dalle‐Donne *et al*. 2003; Ghezzi & Bonetto, 2003). There is a large body of evidence supporting the accumulation of ROS‐induced damaged proteins in senescent cells and tissues from old animals and our previous work has shown that muscles of old wild‐type (WT) mice have an elevated content of a marker of oxidative damage, 3‐nitrotyrosine residues, in the major cytosolic protein carbonic anhydrase III (Vasilaki *et al*. 2007) and increased protein carbonyl content (Broome *et al*. 2006) in comparison with muscles from adult WT mice. Accumulation of oxidised proteins can lead to formation of insoluble protein aggregates, and therefore carbonylated proteins and other irreversibly modified proteins must be degraded in order to prevent them from forming aggregates.

The main players in proteostasis maintenance influencing skeletal muscle are the chaperones, the calcium‐dependent calpains, and the ubiquitin–proteasome and the lysosome–autophagy systems. These components are responsible for the fate of unfolded, misfolded or oxidised proteins, i.e. whether they will refold into their original conformation or whether they will be removed from the cell (Kaushik & Cuervo, 2015; Anthony, 2016; Hohn *et al*. 2017). When a protein is targeted for degradation, chaperones often dictate which proteolytic pathway these unfolded, misfolded or oxidised proteins will follow. Many chaperones are HSPs, which are named according to their molecular mass, e.g. the HSP70 family (which consists of the constitutively expressed HSC70 and highly inducible HSP70) and HSP90, and interventions that maintain overexpression of HSPs, such as HSP70, prevent the accumulation of oxidative damage and preserve some aspects of age‐related muscle dysfunction (McArdle *et al*. 2004; Broome *et al*. 2006).

During chaperone‐mediated autophagy (CMA), substrate proteins are first recognised and bind to heat shock cognate, HSC70. The resulting complex is then targeted to the lysosomes by binding to the lysosomal CMA receptor known as lysosome‐associated membrane protein type 2A (LAMP‐2A), at which point the target protein is unfolded and translocated into the lysosomal lumen for degradation (Cuervo & Wong, 2014; Zhou *et al*. 2017). CMA operates at basal conditions in most mammalian cells, but it is mostly activated in response to stressors, such as oxidative stress (Xilouri & Stefanis, 2016). The activity of CMA has been shown to decline with age in some tissues such as the central nervous system and this decline, which associates with the accumulation of damaged/oxidised/aggregated proteins has been proposed to contribute to tissue dysfunction and possibly to some common age‐related human disorders, such as Parkinson's and Alzheimer's disease (Xilouri & Stefanis, 2016). However, little is known about CMA in other tissues such as skeletal muscle during ageing. Our previous work has shown that oxidative damage to proteins in muscle during ageing is associated with an increase in HSC70 content in quiescent muscles of old mice (Vasilaki *et al*. 2006b) suggesting that muscles of old mice are trying to adapt in order to prevent the accumulation of oxidised proteins. This is in contrast to the findings from a recent study by Zhou and colleagues who demonstrated decreased protein levels of HSC70 and LAMP‐2A in muscle from old mice but an increase in ubiquitinated proteins (Zhou *et al*. 2017). Currently, the function of CMA in skeletal muscle is not well understood and since we did not measure LAMP‐2A or ubiquitinated proteins in our study, we can only speculate that the increased content of HSC70 in muscles of old mice does not result in functional CMA as it may be the case that the formation of damaged proteins overwhelms their degradation and instead contributes to protein aggregation and subsequent loss in proteostasis mechanisms. It is also worth noting that once misfolded proteins organise into oligomers or insoluble aggregates, the only option for their elimination is by degradation in lysosomes via macroautophagy (MA) or expulsion outside the cell by exosomes (Kaushik & Cuervo, 2015). Therefore further studies are required in order to identify the exact mechanisms involved.

### A novel mouse model of frailty: the Cu/ZnSOD knockout mouse

A clear link between age‐related muscle loss and increased ROS production has been indicated by studies of mice lacking Cu/ZnSOD (*Sod1^−/−^* mice). These mice have a significantly shortened lifespan (∼30%) compared to WT mice and have an accelerated decline in muscle mass and function (Muller *et al*. 2006). In addition, adult *Sod1^−/−^* mice show loss of motor function and contractility, declines in nerve conduction, decline in the number of motor units, partial denervation, degeneration of NMJs and increased muscle mitochondrial ROS (mtROS) generation and mitochondrial dysfunction (Flood *et al*. 1999; Shefner *et al*. 1999; Jang *et al*. 2010; Vasilaki *et al*. 2010; Larkin *et al*. 2011; Sims‐Robinson *et al*. 2013; Deepa *et al*. 2017). In contrast to adult WT mice, but in common with old WT mice, adult mice lacking Cu/ZnSOD have an elevated content of 3‐nitrotyrosine residues (Vasilaki *et al*. 2007) and also demonstrate a constitutive activation of NFκB and a constitutive increase in the content of HSPs in muscle at rest (Vasilaki *et al*. 2010).

**Figure 1:**
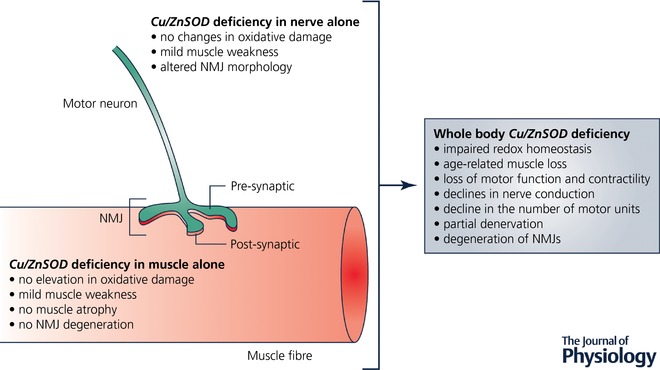
Cu/ZnSOD deficits in either the motor neuron or muscle alone are not sufficient to initiate a full sarcopenic phenotype as seen in *Sod1^−^^/^^−^* mice

The great extent to which changes in *Sod1^−/−^* mice mimic normal ageing indicates that *Sod1^−/−^* mice provide a model in which to study mechanistic links between oxidative stress and sarcopenia and to gain insight into mechanisms of age‐associated atrophy and weakness. While strong associations exist between degeneration of NMJs and declines in mass and force both in *Sod1^−/−^* mice and with normal ageing (Jang & Van Remmen, 2011), knowledge of whether age‐associated muscle wasting and weakness are due to changes proximal or distal to neuromuscular synapses is a major gap in our understanding of sarcopenia. To address questions of the relative importance of pre‐ *vs*. postsynaptic changes, we developed unique mouse models with tissue‐specific targeting of Cu/ZnSOD. These models have generated several key findings. (1) Partial restoration of Cu/ZnSOD only in neurons of *Sod1^−/−^* mice prevented the increases in muscle mtROS production, premature muscle atrophy, and weakness observed in *Sod1^−/−^* mice (Sakellariou *et al*. 2014). In contrast to *Sod1^−/−^* mice, the level of protein nitration and the protein content of a peroxynitrite reductase, peroxiredoxin 5 (PRXV), as well as the contents of key HSPs in skeletal muscle from these mice, were not different from WT levels, indicating no change in the overall redox status. (2) Mice with *Sod1* deficiency in neurons alone (n*Sod1*KO mice) do not show atrophy in gastrocnemius muscles, show only mild weakness and limited evidence of NMJ disruption, and show no significant changes in either mtROS generation or oxidative damage measured by 3‐nitrotyrosine residues suggesting that Cu/ZnSOD deficit in the motor neuron alone is not sufficient to initiate a full sarcopenic phenotype (Sataranatarajan *et al*. 2015). (3) Finally, mice lacking Cu/ZnSOD only in muscle fibres do not show NMJ degeneration or muscle atrophy, show no changes in the HSP content, and oxidative damage is not elevated, but they do show weakness and increased susceptibility to injury (Zhang *et al*. 2013). Collectively, these data suggest that redox homeostasis in motor neurons is a critical factor in initiating sarcopenia, but that the progression of sarcopenia is determined by complex interactions between both pre‐ and postsynaptic factors (Fig. 1).

Because adult mice lacking Cu/ZnSOD reproduce many of the main features seen in old WT mice, they may indicate important mechanisms that lead to loss of muscle fibres and function that are relevant to the ageing of WT mice. The *Sod1^−/−^* mice as well as our other novel mouse models with tissue specific modulation of Cu/ZnSOD have also demonstrated the importance of nerve–muscle interactions in the maintenance of neuromuscular function where ROS homeostasis is altered. Because our data indicate that motor neuron deficits arising from an oxidised redox status are critical in sarcopenia, our future work will focus on determining the impact of oxidative stress in motor neurons on NMJ formation and maintenance and the impact of directly disrupting NMJs on key postsynaptic muscle functions.

In summary, current findings support the hypothesis that increased generation of ROS is an important component of the ageing process, providing a link between accumulation of oxidative damage and muscle dysfunction.

## Additional information

### Competing interests

None

### Author contributions

A.V. produced the manuscript. All authors approved the final version of the manuscript and all persons designated as authors qualify for authorship, and all those who qualify for authorship are listed.

### Funding

The authors would like to thank National Institutes of Health AG051442 for funding. A.R. and H.V.R. are supported by a Senior Research Career Scientist award from the US Department of Veterans Affairs.
